# The electrical heart axis in fetuses with congenital heart disease, measured with non-invasive fetal electrocardiography

**DOI:** 10.1371/journal.pone.0275802

**Published:** 2022-10-20

**Authors:** L. Noben, C. Lempersz, E. R. van den Heuvel, Z. Zhan, F. P. H. A. Vandenbussche, A. B. C. Coumans, M. C. Haak, R. Vullings, S. G. Oei, S. A. B. Clur, J. O. E. H. van Laar

**Affiliations:** 1 Department of Obstetrics and Gynecology, Máxima Medical Center, Veldhoven, The Netherlands; 2 Eindhoven MedTech Innovation Center (e/MTIC), Eindhoven, The Netherlands; 3 Department of Mathematics and Computer Science, Eindhoven University of Technology, Eindhoven, The Netherlands; 4 Department of Obstetrics and Gynecology, Radboud University Medical Center, Nijmegen, The Netherlands; 5 Department of Obstetrics and Gynecology, Maastricht University Medical Center, Maastricht, The Netherlands; 6 Department of Obstetrics and Gynecology, Leiden University Medical Center, Leiden, The Netherlands; 7 Department of Electrical Engineering, Eindhoven University of Technology, Eindhoven, The Netherlands; 8 Department of Pediatric Cardiology, Amsterdam University Medical Centers, Amsterdam, The Netherlands; Texas A&M University College Station, UNITED STATES

## Abstract

**Objectives:**

To determine if the electrical heart axis in different types of congenital heart defects (CHD) differs from that of a healthy cohort at mid-gestation.

**Methods:**

Non-invasive fetal electrocardiography (NI-fECG) was performed in singleton pregnancies with suspected CHD between 16 and 30 weeks of gestation. The mean electrical heart axis (MEHA) was determined from the fetal vectorcardiogram after correction for fetal orientation. Descriptive statistics were used to determine the MEHA with corresponding 95% confidence intervals (CI) in the frontal plane of all fetuses with CHD and the following subgroups: conotruncal anomalies (CTA), atrioventricular septal defects (AVSD) and hypoplastic right heart syndrome (HRHS). The MEHA of the CHD fetuses as well as the subgroups was compared to the healthy control group using a spherically projected multivariate linear regression analysis. Discriminant analysis was applied to calculate the sensitivity and specificity of the electrical heart axis for CHD detection.

**Results:**

The MEHA was determined in 127 fetuses. The MEHA was 83.0° (95% CI: 6.7°; 159.3°) in the total CHD group, and not significantly different from the control group (122.7° (95% CI: 101.7°; 143.6°). The MEHA was 105.6° (95% CI: 46.8°; 164.4°) in the CTA group (n = 54), -27.4° (95% CI: -118.6°; 63.9°) in the AVSD group (n = 9) and 26.0° (95% CI: -34.1°; 86.1°) in the HRHS group (n = 5). The MEHA of the AVSD and the HRHS subgroups were significantly different from the control group (resp. p = 0.04 and p = 0.02).

The sensitivity and specificity of the MEHA for the diagnosis of CHD was 50.6% (95% CI 47.5% - 53.7%) and 60.1% (95% CI 57.1% - 63.1%) respectively.

**Conclusion:**

The MEHA alone does not discriminate between healthy fetuses and fetuses with CHD. However, the left-oriented electrical heart axis in fetuses with AVSD and HRHS was significantly different from the control group suggesting altered cardiac conduction along with the structural defect.

**Trial registration:**

**Clinical trial registration number:** NL48535.015.14.

## 1. Introduction

Congenital heart disease (CHD) is the most common congenital anomaly, with a reported prevalence of 8 per 1000 live births [1–3]. It is a major cause of neonatal morbidity and mortality [1–10]. Prenatal detection of CHD allows for deliberate management to optimize the preoperative neonatal condition and therefore improve neonatal outcome. Furthermore, it keeps the option of pregnancy termination open if the diagnosis is made before the legal limit for pregnancy termination in the said country [11–16].

Screening for CHD is currently performed by means of the second-trimester anomaly scan around 20 weeks of gestation [17]. Since the introduction of national screening programs, the overall detection rate for CHD in low-risk populations has increased up to 50–60% in some countries in Europe [6,8,18–21]. The detection rate is strongly correlated with the severity of the CHD [22]. The highest detection rates are those of univentricular defects such as hypoplastic left heart syndrome and heterotaxy, reaching up to 90% [18,22]. The lowest detection rates are seen in CHD involving the outflow tracts, which are not visible on the four chamber view [22]. Recent evaluation showed that adding the three vessel view as part of the screening program significantly increased detection rates of both tetralogy of Fallot (TOF) and transposition of the great arteries (TGA) [23]. In specialized tertiary care centers with experienced sonographers, the general detection rate of CHD rose to 89% [24]. As only 10% of the infants born with CHD are born to mothers with known risk factors, the majority of these mothers will not have screening in a tertiary care center [25].

We hypothesize that non-invasive fetal electrocardiography (NI-fECG) can play a role in raising detection rates for CHD, primarily in the low-risk population. We previously showed a right-oriented electrical heart axis in healthy fetuses, due to fetal right ventricular dominance as a result of the unique fetal circulation and differential ventricular cardiac output favoring the right ventricle [26]. Structural anomalies in fetuses with CHD may be associated with an abnormal electrical heart axis as is seen postnatally. The objective of this study was to investigate the possibility to detect CHD based on a deviated electrical heart axis.

## 2. Materials and methods

We conducted a multicenter case-cohort study from May 2014 until September 2018 at the following tertiary care hospitals in the Netherlands: Máxima Medical Center Veldhoven, Amsterdam University Medical Center, Radboud University Medical Center Nijmegen, Leiden University Medical Center and Maastricht University Medical Center. The study protocol was approved by the institutional review board of the Máxima Medical Center, Veldhoven, the Netherlands (NL48535.015.14). Written informed consent was obtained prior to enrolment.

### 2.1 Study population

Women pregnant with a fetus suspected for CHD, based on advanced ultrasound evaluation, were asked to participate in this prospective cohort study. Women 18 years or older and pregnant of a singleton between 16 and 30 weeks of gestation were included. In addition, measurements of fetuses who were included in our previously published healthy cohort and diagnosed with CHD postpartum were transferred to the CHD cohort [27,28].

Exclusion criteria were a fetal cardiac arrhythmia and insufficient understanding of the Dutch language.

The following data were gained prospectively: general medical history, maternal gravidity and parity, obstetrical history, gestational age at inclusion, suspected CHD based on fetal echocardiography. Postpartum, neonatal charts were checked for confirmation of the CHD through echocardiography by a pediatric cardiologist. If the pregnancy was terminated immaturely, post mortem examination reports were consulted if available.

### 2.2 Measurements

**Fig 1:**
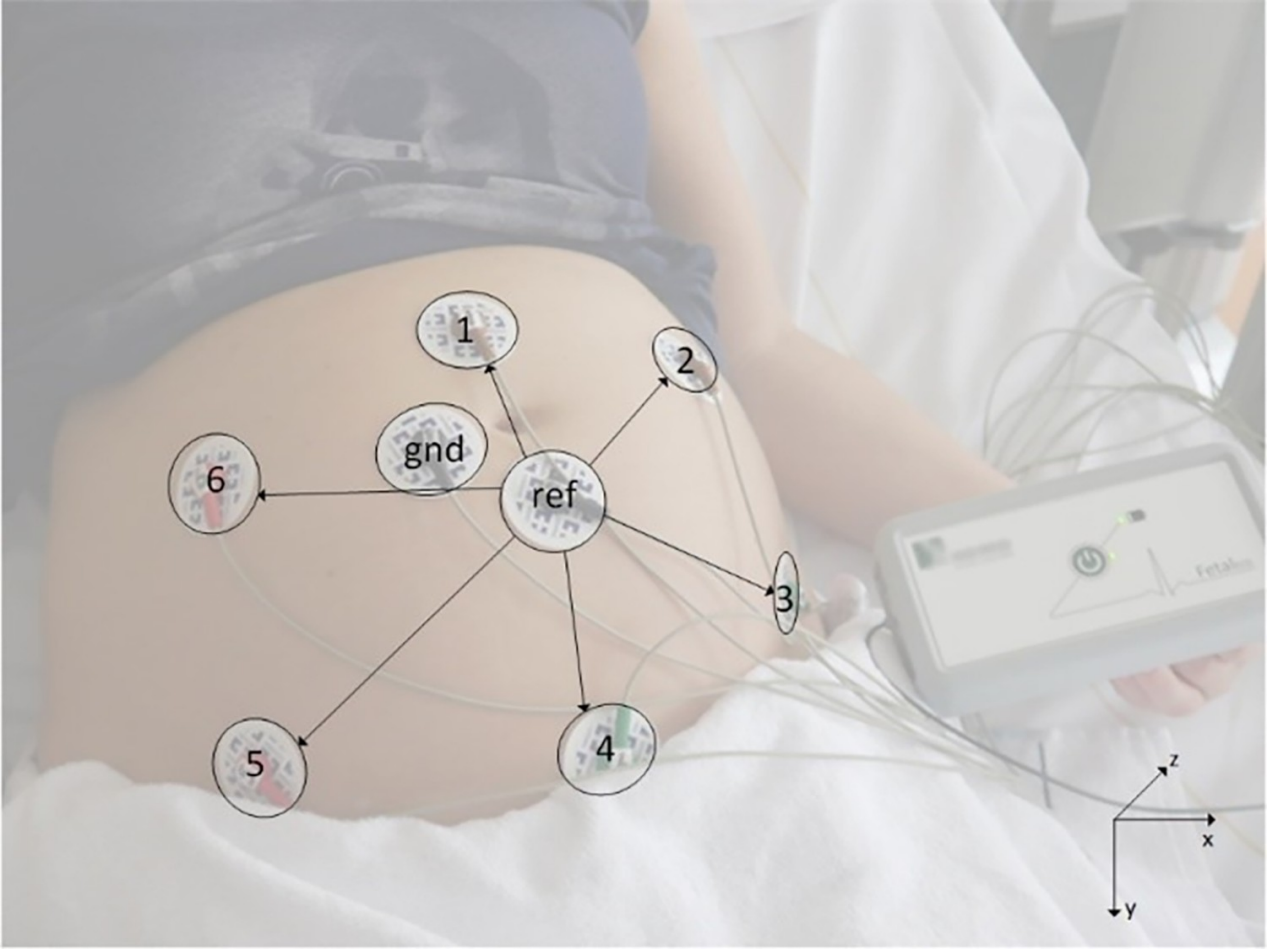
The non-invasive fetal electrocardiogram (NI-fECG) setup with electrodes on the maternal abdomen.

Fetal ECG measurements were performed using a prototype fetal ECG system (Nemo Healthcare BV, the Netherlands) after a fetal echocardiographic examination in a tertiary care center. Pregnant women were positioned in a semi-recumbent position to prevent aortocaval compression. Eight adhesive Ag/AgCl electrodes (Red DotTM, 3M Health Care, Ontario, Canada) were placed on the abdomen in a fixed configuration in order to yield six channels of bipolar electrophysiological measurements. Two electrodes functioned as common ground and reference electrode respectively (Fig 1). Before applying the electrodes, the abdominal skin was washed with water and soap and then scrubbed using medical abrasive paper (Red DotTM Trace Prep, 3M Health Care, Ontario, Canada) to optimize skin impedance. Each measurement lasted around 40 minutes. The position of the fetus was determined by ultrasonography at four fixed time intervals during the measurement.

The recordings were digitized at 500Hz sampling frequency and stored on a computer for offline analysis. This offline analysis consisted of a series of signal processing steps, designed to suppress interferences and standardize the fetal ECG signals for fetal orientation, so that the fetal electrical heart axis could be measured. These signal processing steps have been described in more detail in [29]. In the first step of signal processing, interferences from maternal ECG, abdominal muscles, and extracorporal sources were suppressed by an adaptive template-based method [30]. As a result, for each of the six recorded signals a fetal ECG signal is obtained, yet at relatively low signal-to-noise ratio. Because each fetus could have a different orientation with respect to the maternal abdomen and the recording electrodes placed on the abdomen, the fetal ECG signals could be different between participants, but also within participants due to fetal movement.

The second step in the signal processing aimed to standardize for fetal orientation. To allow for such standardization, first for every heartbeat a fetal vectorcardiogram was calculated, combining the information from the six abdominal signals into a 3-dimensional fetal ECG complex [31]. This vectorcardiogram could subsequently be tracked over time, detecting fetal movements and correcting for them by rotating the fetal vectorcardiogram in 3-dimensional space. Finally, another rotation in 3-dimensional space was applied that corrected for the fetal orientation. For instance, if the ultrasound indicated that the fetus was in cephalic position, the recorded fetal vectorcardiogram was rotated by 180 degrees to represent the fetal vectorcardiogram as if the fetus was in breech position, mimicking the anatomical position. Similarly, a fetal back which was oriented to the maternal abdomen was rotated along the longitudinal axis as if the fetal back was oriented to the maternal spine. The parts of the measurements of sufficient signal quality, closest to the performance of the ultrasound determining fetal orientation, were used to create the vectorcardiogram.

Finally, to enhance the signal-to-noise ratio, orientation-standardized fetal vectorcardiograms were averaged over multiple heartbeats to yield one fetal vectorcardiogram per measurement.

The orientation of the electrical heart axis was defined as the direction in which the vectorcardiogram had its maximum amplitude [32]. The latter direction was estimated as the average direction of the dominant vectors in the QRS complex, defined as the vectors from the point that the R-wave exceeded 70% of its maximum value until the point that it fell below 70% of the maximum value. The orientation of the fetal heart axis was expressed in degrees ranging from minus 180° to plus 180° and calculated in the frontal plane, where minus 90° is located superiorly.

### 2.3 Classification of CHD

CHD were classified in subgroups based on the type of defect and its hemodynamic consequences. Table 1 shows an overview of all included CHD types and their corresponding subgroup. We included the following three CHD (subgroups) for statistical analysis: conotruncal anomalies (CTA), atrioventricular septal defects (AVSD) and hypoplastic right heart syndrome (HRHS). These were chosen for the following reasons. CTA make up an important part of all CHD and may be missed on the fetal anomaly scan, especially when the outflow tracts are difficult to image due to fetal position and the complex multiplanar evaluation, since the four chamber view may appear normal. Furthermore, some fetuses with undiagnosed CTA, such as transposition of the great arteries (TGA) with intact septum or pulmonary atresia with ventricular septal defect (extreme tetralogy of Fallot [TOF]), may develop acute hypoxia in the first few days postpartum when the arterial duct undergoes physiological closure. Without immediate intervention, i.e. administration of prostaglandins to keep the arterial duct open, this can be a life-threatening event. We expected the fetal ECG to show a right axis.

Both fetuses with AVSD and HRHS may be expected to have a left-oriented electrical heart axis. We chose to include these CHD where the most overt differences in electrical heart axis can be expected compared to the healthy control group, since literature on the electrical heart axis in fetuses with CHD is scarce [16,33,34].

**Table 1:** Distribution of the different types of CHD included in the study population.

CHD group	n	CHD type		n	GA at measurement^$^	% of study population
*Overall*	*127*	*All*			*23*.*2 ± 3*.*2*	*100*
Septal defects	25				23.28 ± 3.2	19.7
		VSD		16	23.4 ± 3.5	12.6
		AVSD		9	23.1 ± 2.7	7.1
Conotruncal anomalies	54				23.2 ± 3.6	42.5
		TGA (IVS and VSD)		27	23.6 ± 3.2	21.3
			TGA + IVS	19	24.1 ± 3.2	15.0
			TGA + VSD	8	21.7 [20.2–23.5]	6.3
		TOF		16	23.2 ± 2.9	12.6
		VSD + pulmonary atresia		2	20.7; 23.1	1.6
		DORV + pulmonary stenosis		2	19.4; 21.3	1.6
		TGA + VSD + pulmonary stenosis		2	19.9; 24.3	1.6
		Truncus arteriosus		1	22.1	1.0
		ccTGA		2	26.0; 28.3	1.6
		DORV, no PS		2	20.4; 20.6	1.6
Single ventricle	10				20.6 [20.0–24.0]	
		Hypoplastic right heart syndrome		5	20.4 [19.9–21.9]	3.9
		Hypoplastic left heart syndrome		5	23.1 ± 4.8	3.9
Complex	15				20.9 [20.1–21.7]	
		AVSD combined with other cardiac anomalies		3	20.8 ± 4.0	2.4
		DILV		4	20.5 ± 0.6	3.1
		Ebstein anomaly		5	20.7 [20.4–27.0]	3.9
		Other		3	21.0; 21.1; 23.7	2.4
Miscellaneous	5				25.8 ± 2.4	3.9
R/L disproportion	9				26.2 ± 2.9	
		Aortic coarctation		8	25.9 ± 3.0	6.3
		No aortic coarctation		1	28.3	1.0
Vascular ring	6				21.9 ± 1.5	4.7
Chromosomal aberration		Noonan syndrome		3	19.4; 21.0; 28.0	2.4

^$^ Data provided are percentages or mean ± SD. Median [interquartile range] are provided for variables that are not normally distributed, Individual values are shown when n was low.

Abbreviations: AVSD = atrioventricular septal defect, ccTGA = congenitally corrected transposition of the great arteries (double discordance), CHD = congenital heart disease, DILV = double inlet left ventricle, DORV = double outlet right ventricle, IVS = intact ventricular septum, TGA = transposition of the great arteries, TOF = tetralogy of Fallot, VSD = ventricular septal defect.

### 2.4 Statistical analysis

Results from our CHD cohort were compared using our previously published cohort of healthy fetuses as reference group [27,28]. Descriptive statistics were used to determine baseline characteristics of our overall CHD cohort. Differences in baseline characteristics between the overall CHD group and the healthy control group were tested using the Mann-Whitney U test for not normally distributed data and an independent T-test for normally distributed data.

Spherical statistics were applied to compare the two-dimensional mean electrical heart axis (MEHA) in the frontal plane between the groups, which required using the individual Cartesian coordinates. The observed frontal angle was determined in the (x,y)-plane, where x represented the left-right horizontal axis and y represented the craniocaudal axis. Since the length of the vector of the electrical heart axis in the frontal plane is influenced by electrical propagation in all directions, the vector of each fetus was normalized to create unit vectors i.e. with equal length. The normalized coordinates ($\tilde{x},\tilde{y}$) of these unit vectors were calculated as the division of the originate coordinates (x, y) by their Euclidean norm $\sqrt{x^{2}+y^{2}}$.

Descriptive statistics (median with interquartile range (IQR)) were calculated based on the normalized ($\tilde{x},\tilde{y}$) Cartesian coordinates for the overall CHD group as well as for each of the three selected CHD subgroups. Differences between the overall CHD group as well as each CHD subgroup and the control group were tested using the Kolmogorov-Smirnov test.

The mean frontal angle with 95% confidence intervals (CI) were calculated for both the overall CHD group and each CHD subgroup [35].

A likelihood ratio test (LRT) was used to determine differences in frontal angles between the previously published control group and the overall CHD group assuming equal concentration parameters (i.e. similar to equal variances in 2-sample t-tests) [36]. This assumption was verified with a circular concentration test [36]. If the equal concentration assumption was violated, a sensitivity analysis using the non-equal concentration approach suggested by Mardia and Jupp (2000) was performed [36].

Furthermore, a LRT was also performed to determine the overall difference in frontal angles of the CHD subgroups and the control group. In addition, a spherically projected multivariate linear (SPML) regression model with the frontal angle as the outcome and the subgroup as a categorically independent variable (control group was considered as the reference level) was fitted to the data, under the assumption that the data follows a von Mises-Fisher distribution (analogous to the normal distribution in linear regression) [37,38].

Circular discriminant analysis was performed on the unit vectors between the healthy control group and the overall CHD group [39]. Sensitivity and specificity were calculated based on 1000 Monte Carlo cross validation samples (20% of the original sample was randomly selected as the testing sample and the rest used as training sample).

Descriptive statistics were used to describe baseline characteristics, using IBM SPSS statistics version 25.0 (SPSS Inc., Chicago, Ill., USA). Statistical analysis was conducted with SAS (version 9.4, SAS Institute Inc., NC, USA) and R (version 3.5.3, R Foundation, Vienna, Austria). Significance level for all tests was set at 0.05.

## 3. Results

**Fig 2:**
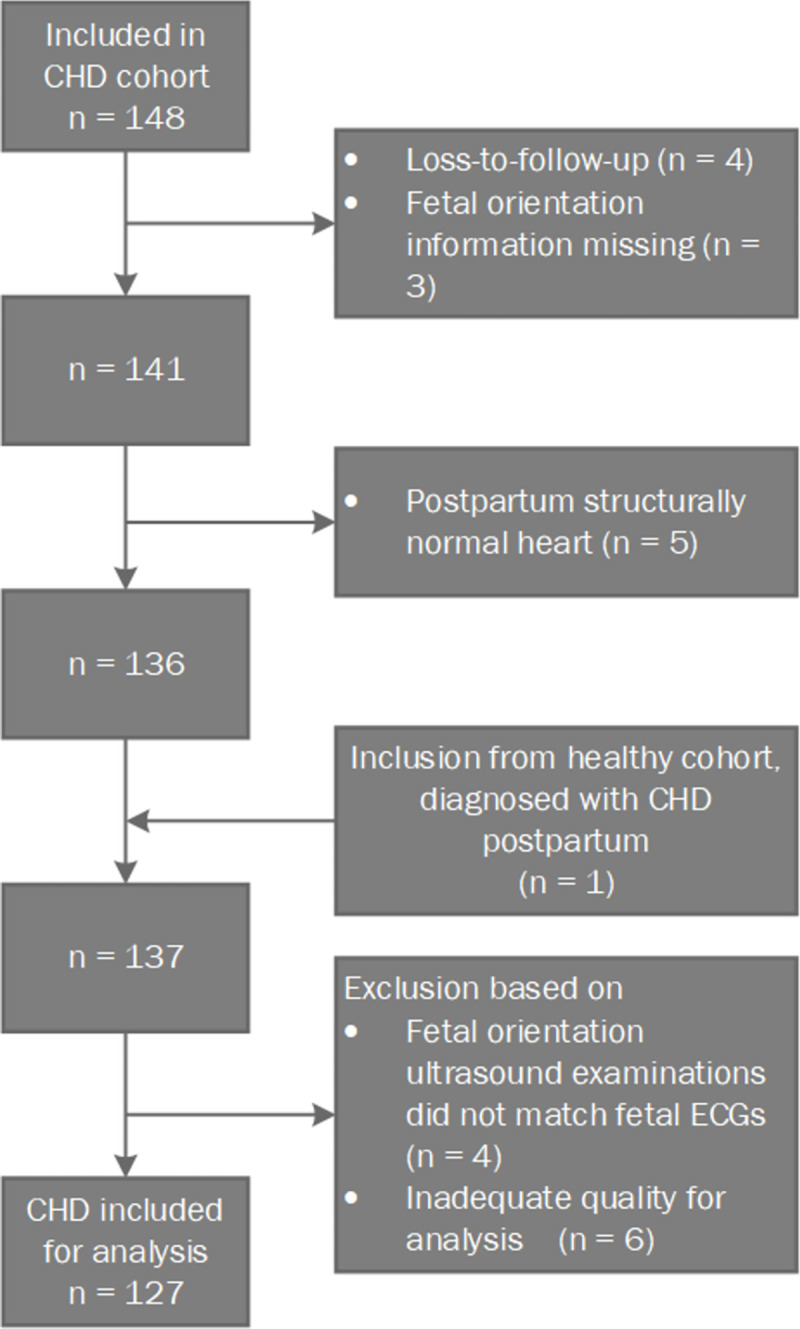
Flow diagram of patient inclusion.

A total of 148 women were included carrying a fetus with suspected CHD after fetal echocardiography. The inclusion process is depicted in Fig 2. The electrical heart axis was determined in 127 fetuses with CHD. Within the overall CHD group, 54 fetuses were allocated to the CTA group, 9 to the AVSD group and 5 to the HRHS group. Table 1 shows an overview of all included CHD types and their corresponding subgroup. Baseline characteristics are shown in Table 2. The CHD group was not different to the normal control group for maternal age, parity or maternal BMI. The gestational age during the NI-fECG measurement for the control group was on average three weeks earlier than for the CHD group (p = 0.00).

**Table 2:** Baseline characteristics of participants.

	CHD		Healthy cohort		p-value
		n		n	
**Maternal Age (years)**	30.5 ± 4.6	127	31.0 [26.0–36.0]	281	0.09^a^
**GA (weeks) at time of measurement** **CTA** **AVSD** **HRHS**	23.2 ± 3.2 23.2 ± 3.6 23.1 ± 2.7 20.8 ± 1.3	127 54 9 5	20.2 ± 1.3	281	0.00^b^
**Nulliparous (%)**	44.1	127	52.0	281	0.14^c^
**BMI (kg/m** ^**2**^ **)**	23.8 [18.4–29.2]	125	22.8 [16.7–28.9]	280	0.07^a^

Data provided are means ± SD. Median [interquartile range] are provided for variables that are not normally distributed. Differences in baseline characteristics between the CHD group and the healthy cohort were tested using the ^a^ Mann-Whitney U test, ^b^ Independent T-test and ^c^ Chi square test.

Abbreviations: AVSD = atrioventricular septal defect, BMI = body mass index, CHD = congenital heart disease, CTA = conotruncal anomaly, GA = gestational age, HRHS = hypoplastic right heart syndrome, kg = kilograms, m = meter.

No significant difference in distribution of the normalized $\tilde{x}$ and $\tilde{y}$ coordinates were found between the overall CHD group and the control group and between each CHD subgroup and the control group (Table 3).

**Table 3:** Summary statistics (median [IQR]) on the two dimensions for the overall CHD group and each subgroup compared to the healthy control group.

Groups									
	Healthy control group n = 281	Overall CHD n = 127		CHD subgroups					
				CTA n = 54		AVSD n = 9		HRHS n = 5	
	Median (IQR)	Median (IQR)	p-value	Median (IQR)	p-value	Median (IQR)	p-value	Median (IQR)	p-value
$\tilde{x}$	-0.35 (1.66)	-0.01 (1.75)	0.22	-0.18 (1.72)	0.78	0.88 (1.49)	0.13	0.63 (0.60)	0.08
$\tilde{y}$	-0.33 (0.96)	-0.24 (0.90)	0.17	-0.33 (1.12)	0.90	0.12 (1.11)	0.10	-0.31 (0.58)	0.90

P-values calculated by means of a Kolmogorov-Smirnov test showed no significant difference in distribution between the overall CHD group as well as each CHD subgroup with respect to the control group on both normalized coordinates.

*Abbreviations*: AVSD = atrioventricular septal defect, CTA = conotruncal anomaly, HRHS = hypoplastic right heart syndrome, IQR = interquartile range.

We previously described reference ranges using 90% prediction intervals for the electrical heart axis in healthy fetuses, based on data from 281 fetuses between 18 and 24 weeks of gestation [28]. The mean frontal angle for this control group was determined at 122.7° (95% CI: 101.7°; 143.6°).

**Fig 3:**
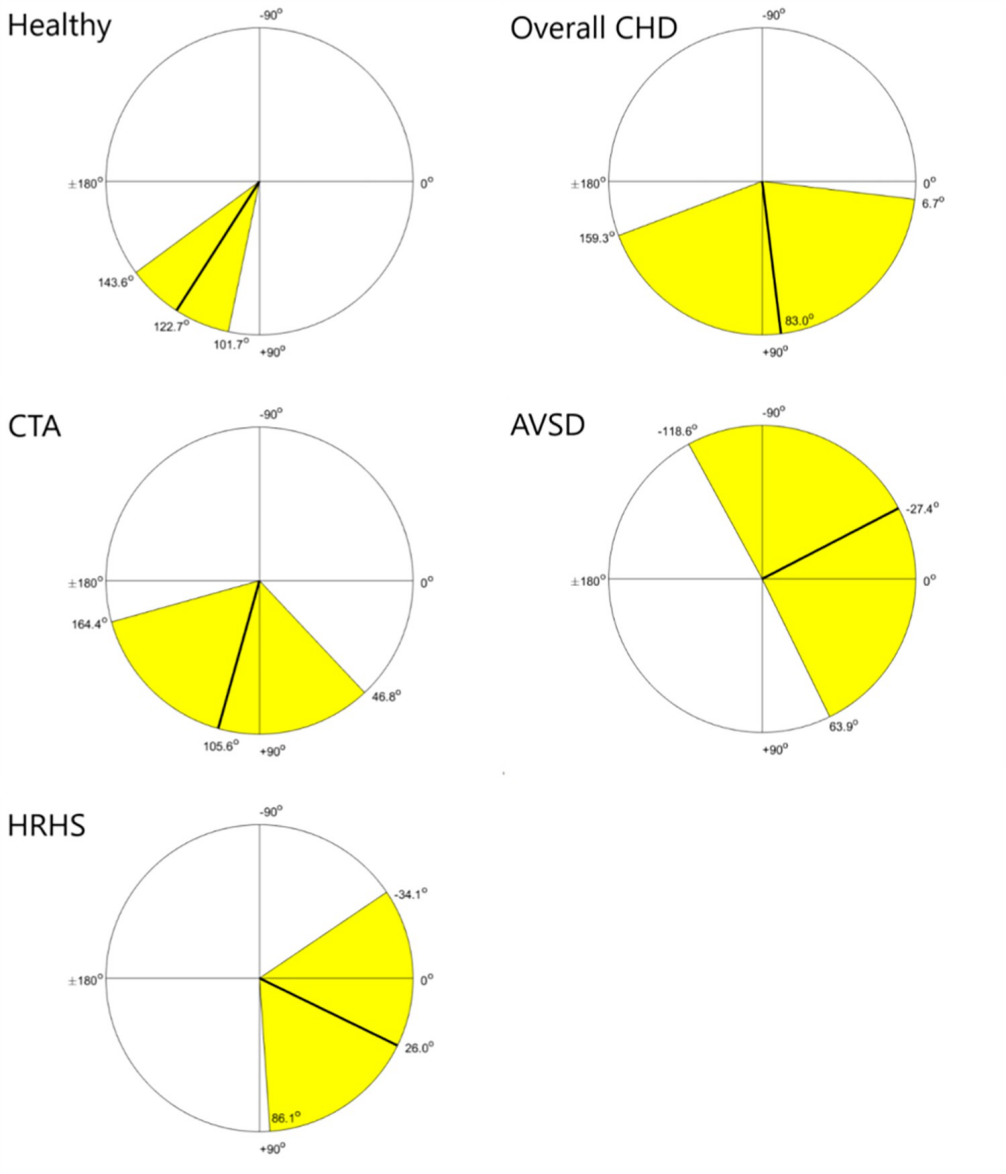
Mean electrical heart axis (MEHA) with corresponding 95% CI in the frontal plane plotted in a circle diagram for each group. Abbreviations: AVSD = atrioventricular septal defect, CHD = congenital heart disease, CTA = conotruncal anomalies, HRHS = hypoplastic right heart syndrome.

In our overall CHD group the mean frontal angle was determined at 83.0° (95% CI: 6.7°; 159.3°). For the three CHD subgroups, the mean frontal angles were estimated at 105.6° (95% CI: 46.8°; 164.4°) for the CTA, -27.4° (95% CI: -118.6°; 63.9°) for the AVSD, and 26.0° (95% CI: -34.1°; 86.1°) for the HRHS group. Fig 3 shows the mean frontal angle with corresponding 95% CI of these groups on a circle diagram.

We found no significant difference in electrical heart axis between the overall CHD group and the healthy control group (test statistic = 2.17, p = 0.14). Since the test for equality of concentration between both groups was significant (test statistic = 3.99, p = 0.046), we conducted a sensitivity analysis which confirmed that there was no difference in electrical heart axis between both groups (test statistic = 1.22, p = 0.27).

Discriminant analysis between the healthy control group and the overall CHD showed a sensitivity of 50.6% (95% CI 47.5% - 53.7%) and a specificity of 60.1% (95% CI 57.1% - 63.1%) for the detection of CHD.

We found a significant difference in electrical heart axis when comparing the healthy control group with all three CHD subgroups (test statistic = 8.35, p = 0.04) with equal concentration across the groups (equal concentration test statistic = 0.62, p = 0.89), indicating a difference in electrical heart axis between these groups. To gain more insight into the difference between each CHD subgroup and the healthy control group, a SPML regression analysis was performed and the results are displayed in Table 4. We found a significant difference in frontal angle between the healthy control group and both the AVSD subgroup (p = 0.04) and the HRHS subgroup (p = 0.02).

**Table 4:** Difference in normalized ($\tilde{x},\tilde{y}$) coordinates between the healthy control group and each CHD subgroup.

	$\tilde{x}$		$\tilde{y}$	
	Estimate (S.E)	p-value	Estimate (S.E)	p-value
Intercept	-0.28 (0.07)	<0.001	-0.36 (0.07)	<0.001
CTA vs healthy	0.17 (0.17)	0.32	0.03 (0.16)	0.86
AVSD vs healthy	0.84 (0.41)	**0.04**	0.53 (0.37)	0.15
HRHS vs healthy	1.29 (0.54)	**0.02**	-0.19 (0.52)	0.72

P-values are obtained by means of a spherically projected multivariate linear (SPML) regression analysis with the frontal angle as the outcome and the subgroup as a categorical independent variable. The healthy control group was considered as reference level. Significant results are shown in bold.

Abbreviations: AVSD = atrioventricular septal defect, CTA = conotruncal anomaly, HRHS = hypoplastic right heart syndrome, S.E. = standard error.

## 4. Discussion

### Main findings

To our knowledge this is the first study of NI-fECGs in a large cohort of fetuses with CHD, looking at the MEHA in the frontal plane. We found no significant difference in MEHA between the healthy control group and the overall CHD group, which resulted in low sensitivity and specificity of the electrical heart axis for the detection of CHD. The MEHA of the AVSD and HRHS subgroups were left-oriented and statistically significant from the healthy control group which may be helpful in the prenatal detection of these types of CHD.

### Interpretation of findings and comparison with existing literature

We previously described a right-oriented MEHA in healthy fetuses around mid-gestation [28]. This right-oriented axis is still present after birth, but gradually deviates towards the left during the first year of life [40]. These changes reflect the developmental changes from fetus to child where the right ventricle is dominant prenatally pumping a higher cardiac output against high resistance in the fetus, and the dominant left ventricle pumping against high resistance in the child and adult. As the pulmonary vascular resistance declines postnatally the workload of the right ventricle is reduced relative to the left ventricle with an associated change in relative ventricular muscle mass [26,41].

We found a MEHA in our overall CHD group which is oriented slightly to the left and not significantly different from that of our healthy control group (χ^2^(df = 1) = 2.17, p = 0.14). Since we included all types of CHD, it comprised a heterogenous group. As this heterogeneity may have confounded our results, we also looked at three clinically relevant subgroups and compared them with the healthy control group as well.

First, we chose the CTA subgroup which makes up a large part of all CHD. The prevalence of CTA varies between prenatal (2.5–21%) and postnatal (10–12%) series [4,42–44] and is influenced by differing prenatal CHD detection rates between countries [45–50]. CTA comprised 42.5% of all CHD included in our study. As the four-chamber view of the heart in many cases of CTA such as TOF and TGA may be normal, detection rates can be improved by using the outflow tract and three vessel views as part of the fetal anomaly scan for CHD screening [8,23,51]. We found a right-oriented MEHA in our CTA subgroup, which was not significantly different from the healthy control group. This was in line with our expectations, since this subgroup comprises mainly fetuses with TOF and TGA, and a right axis deviation is seen postnatally in these defects due to right ventricular hypertrophy and strain analogous to the fetal situation.

Second, we compared the AVSD group with our healthy control group. Only 2 cases describing the electrical heart axis in AVSD fetuses are available in the literature, with inconsistent results [33,34]. We expected to find a distinctly left-orientated MEHA in these fetuses, as is seen in neonates with these defects. Left ventricular hypertrophy may contribute to the deviated electrical heart axis in AVSD [52], but anatomic displacement of the left ventricular (LV) papillary muscles (PM) is more important in the altered electrical activation in this condition [53]. The insertion place of the PM on the ventricular wall coincides with the end of the left bundle branch fascicles. In AVSD, the anterior PM is positioned relatively closer to the septum than the posterior PM which produces a delay in activation of the anterior LV free wall, causing a left anterior hemiblock. Our data confirm a left-oriented MEHA in our AVSD subgroup, which was significantly different from the healthy control group (test statistic = 0.84, p = 0.04). Third, we included fetuses with HRHS. In HRHS there is underdevelopment of the right-sided cardiac structures and thus a relative dominance of the left-sided cardiac musculature, and an expectation of a left-oriented electrical heart axis. Our findings confirm this left-oriented MEHA in our HRHS subgroup, which is significantly different from the healthy control group. (test statistic = 1.29, p = 0.02).

### Strengths and limitations

A major strength of our study is the large cohort of healthy fetuses (n = 281) and fetuses with CHD (n = 127). As the cohort of CHD was heterogenous, the numbers per individual CHD type were small precluding individual analysis per diagnosis. We thus chose for three groups which are prenatally relevant, either due to prevalence or expected abnormal heart axis.

The number of excluded recordings due to inadequate data quality was low (n = 6). However, the NI-fECG technology is currently limited by the lack of real-time results. Offline analysis of the recordings is still required. Automatization of the signal processing steps is ongoing for future implementation in the measurement hardware to address this problem.

The gestational age at time of measurement was three weeks later in the CHD group compared to the healthy control group. As there is limited data available on the course of the electrical heart axis in fetuses during pregnancy, this may have influenced our results. The MEHA of term babies is 110°, ranging from 30° to 180° [54]. This suggests a minimal shift of the electrical heart axis to the left between mid-gestation and term. Therefore, we do not expect this difference in gestational age to have significantly influenced our results.

Before implementation of the NI-fECG technology for the purpose of detecting CHD in a clinical setting can occur, more research is needed in determining which ECG characteristics can accurately differentiate between healthy fetuses and fetuses with a CHD.

### Clinical and research implications

NI-fECG is a patient-friendly method which requires minimum training for healthcare personnel to apply. With further development of the technology, it could be a non-expensive diagnostic test. Our data show that the electrical heart axis in the frontal plane as a single parameter, measured with NI-fECG, does not discriminate between healthy fetuses and fetuses with CHD. However, the left-oriented MEHA in fetuses with AVSD and HRHS differs significantly from the healthy control group suggesting altered cardiac conduction along with the structural defect. Other ECG characteristics such as ECG morphology and cardiac time intervals may unveil information necessary to distinguish fetuses with CHD. More research is needed to evaluate if the addition of a fetal ECG to current prenatal screening increases CHD detection rates.

## Conclusion

The MEHA in our CHD cohort was oriented slightly to the left and not significantly different from that of our healthy control group. Consequently, the sensitivity and specificity of the electrical heart axis in the detection of CHD was low. The MEHA in the AVSD and HRHS subgroups was oriented to the left and significantly different from our healthy control group. More research is needed to see if other ECG characteristics can play a role in the detection of CHD in the future.
